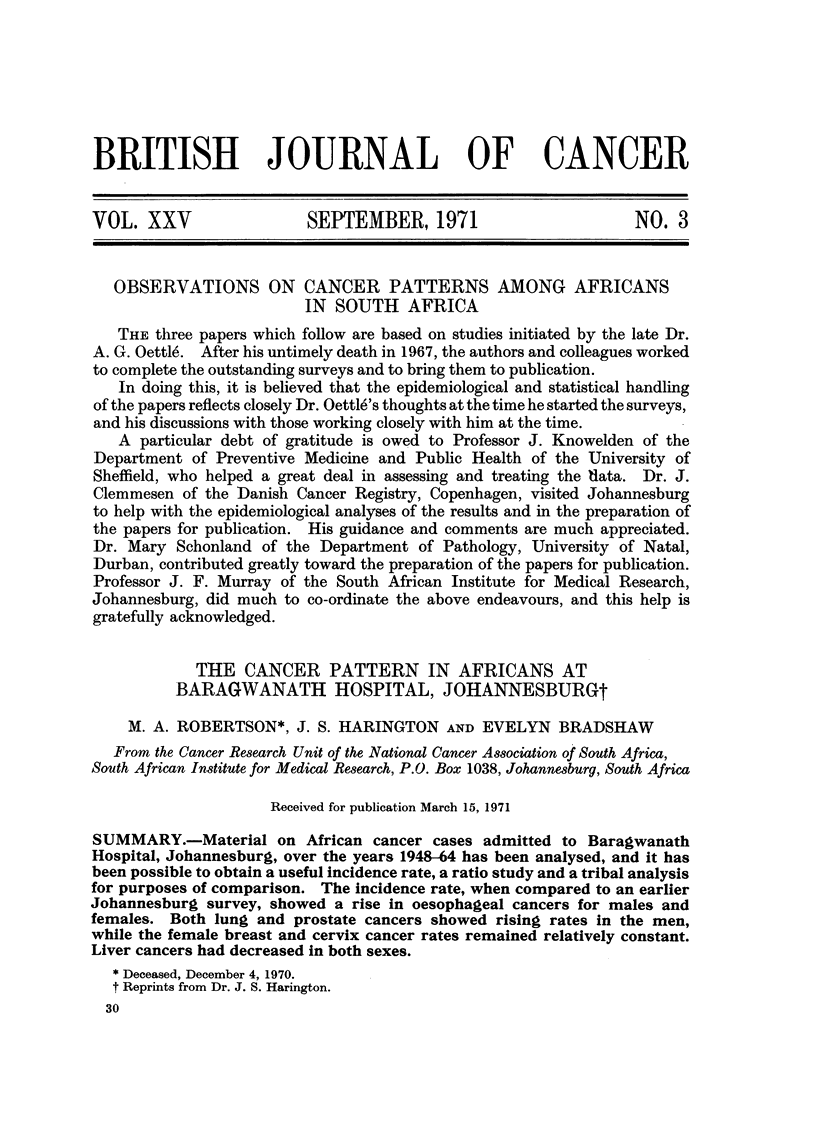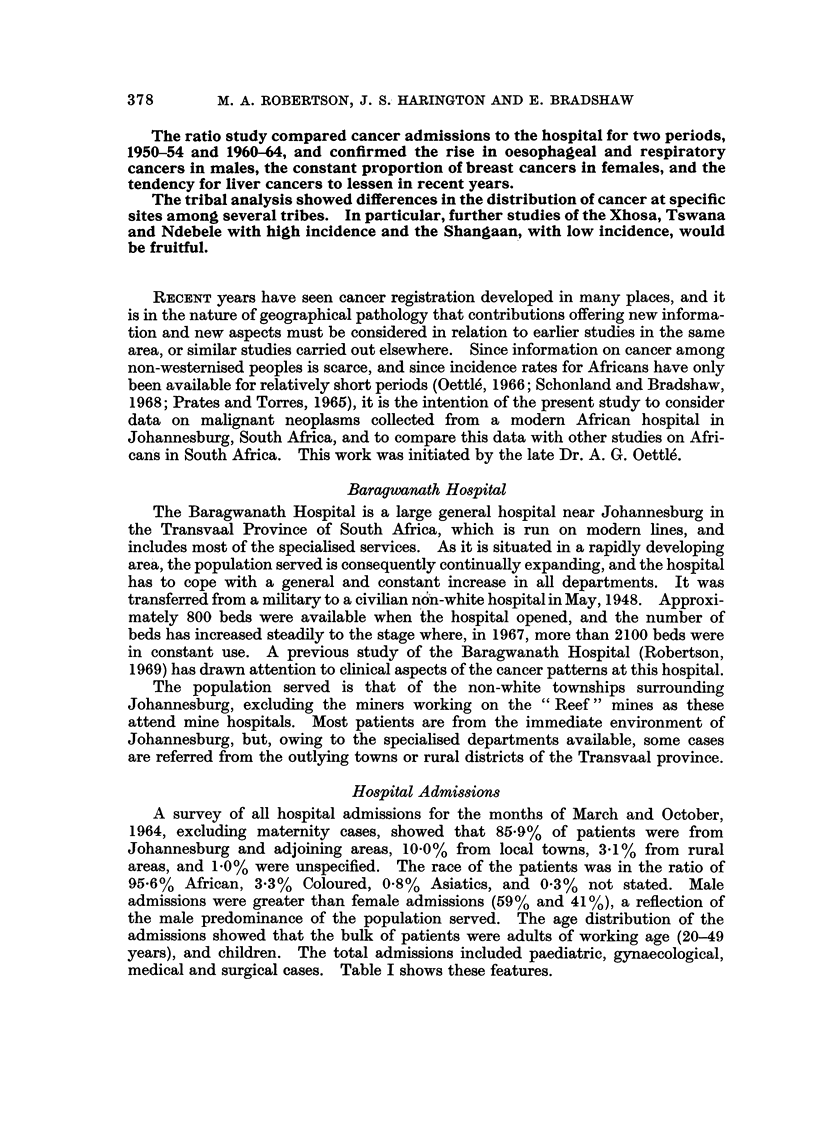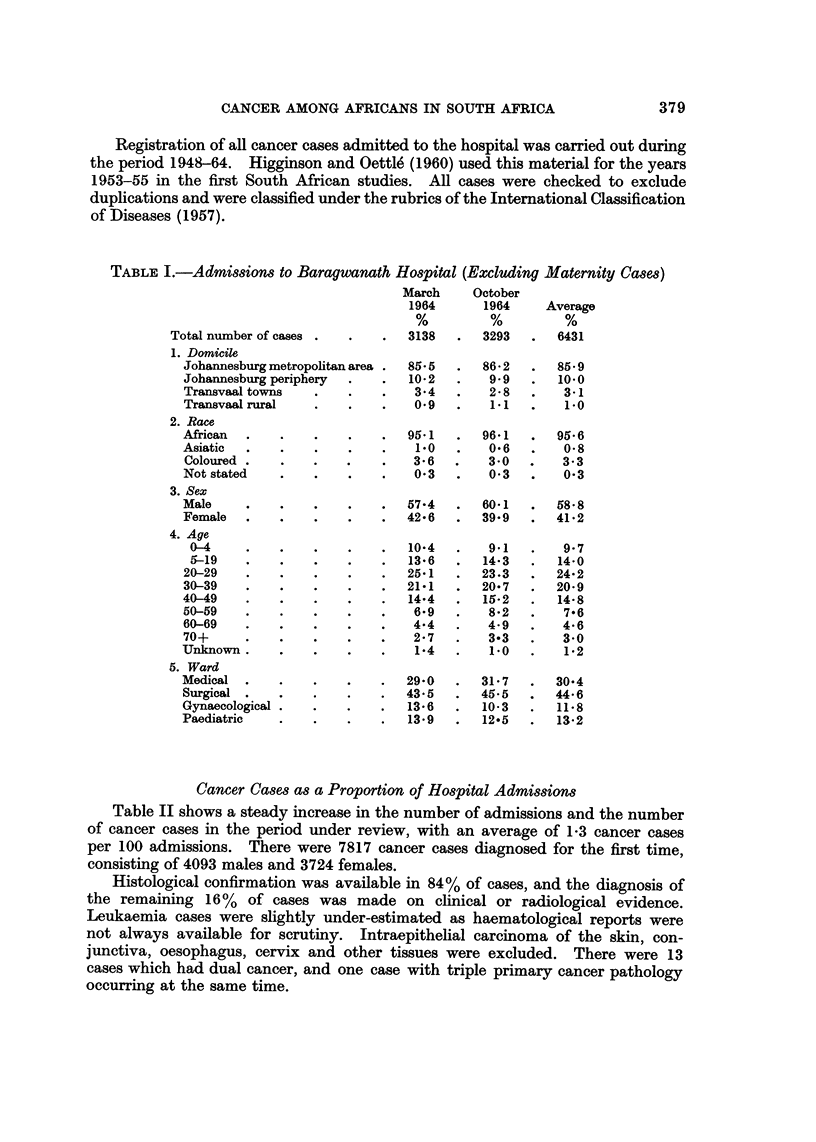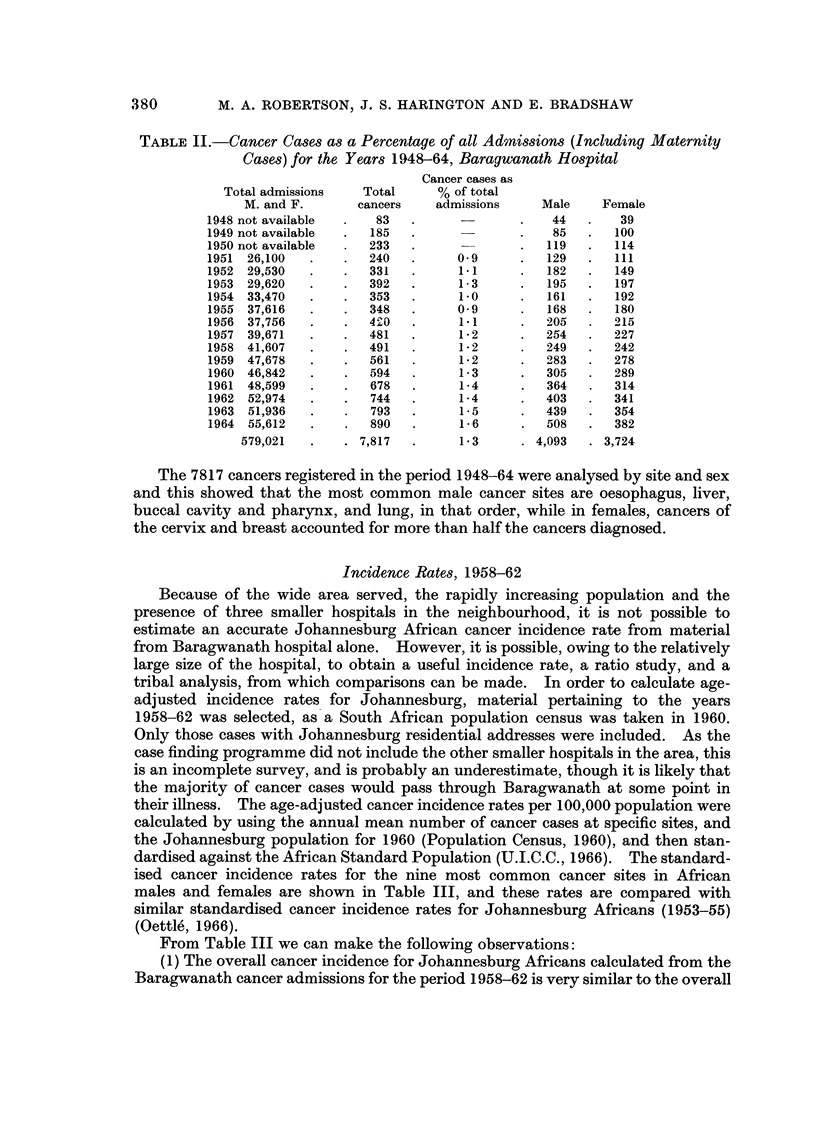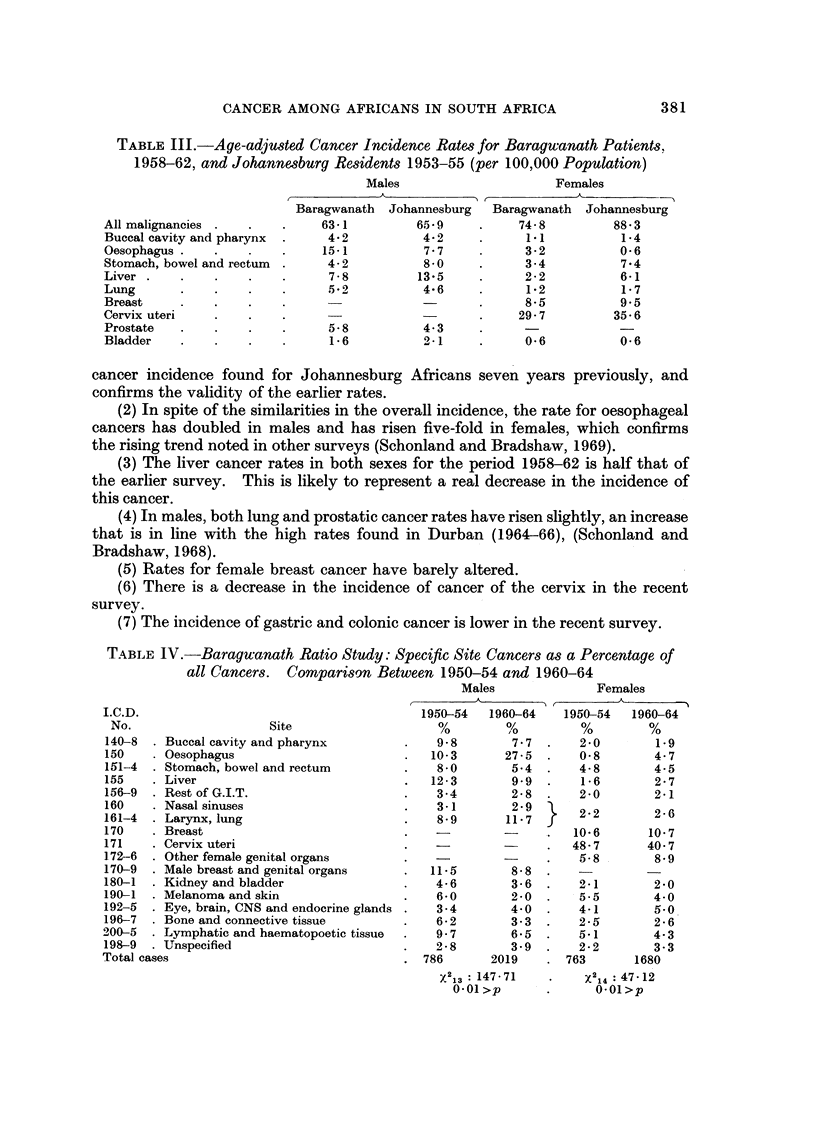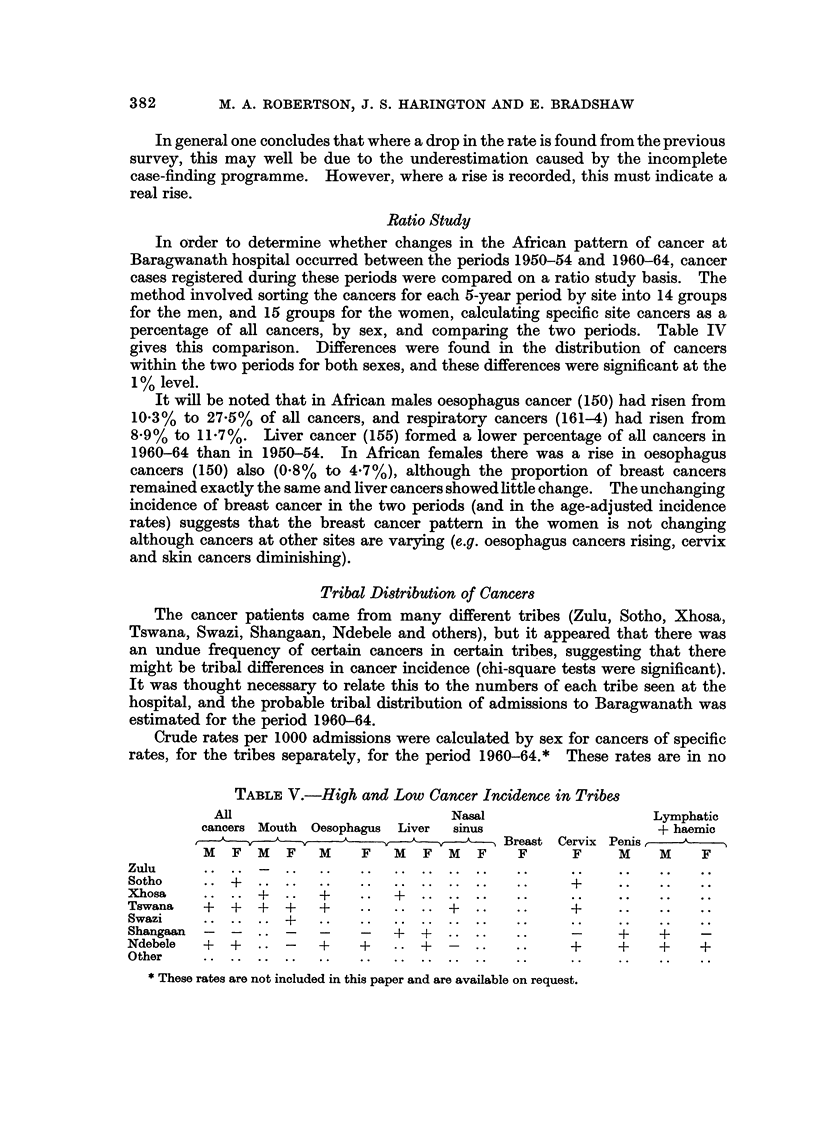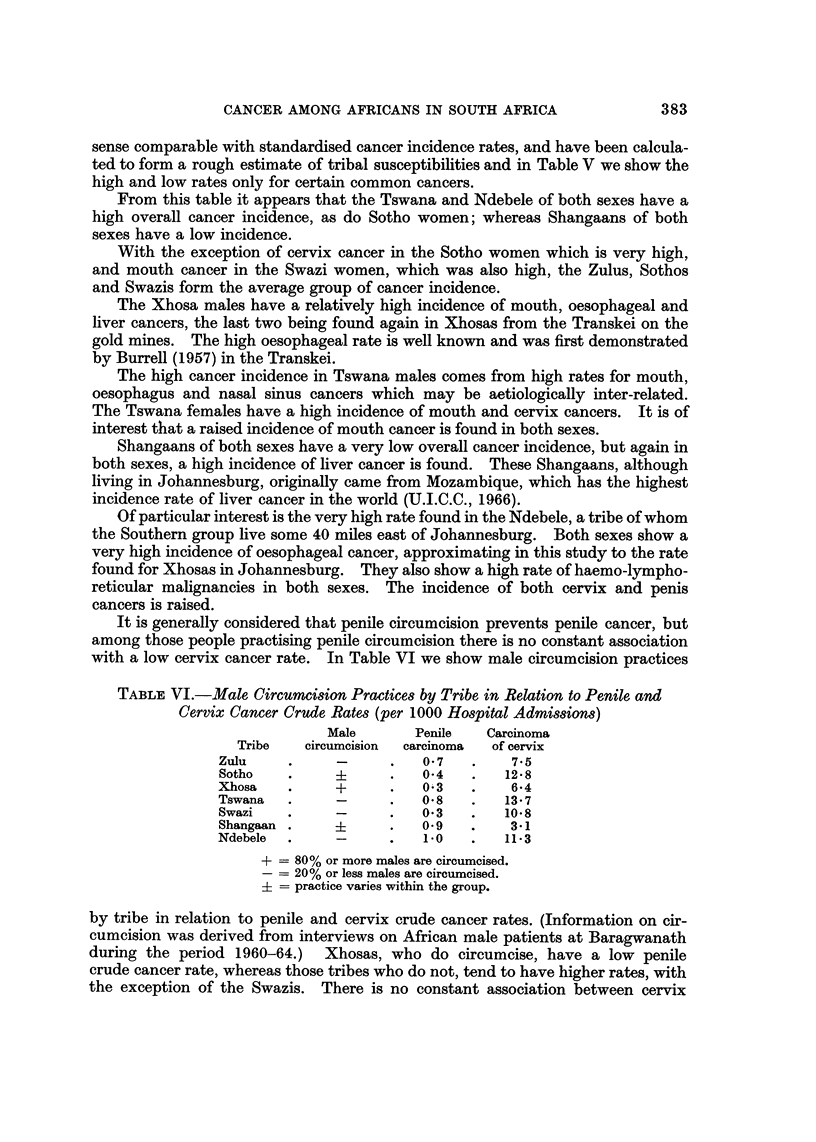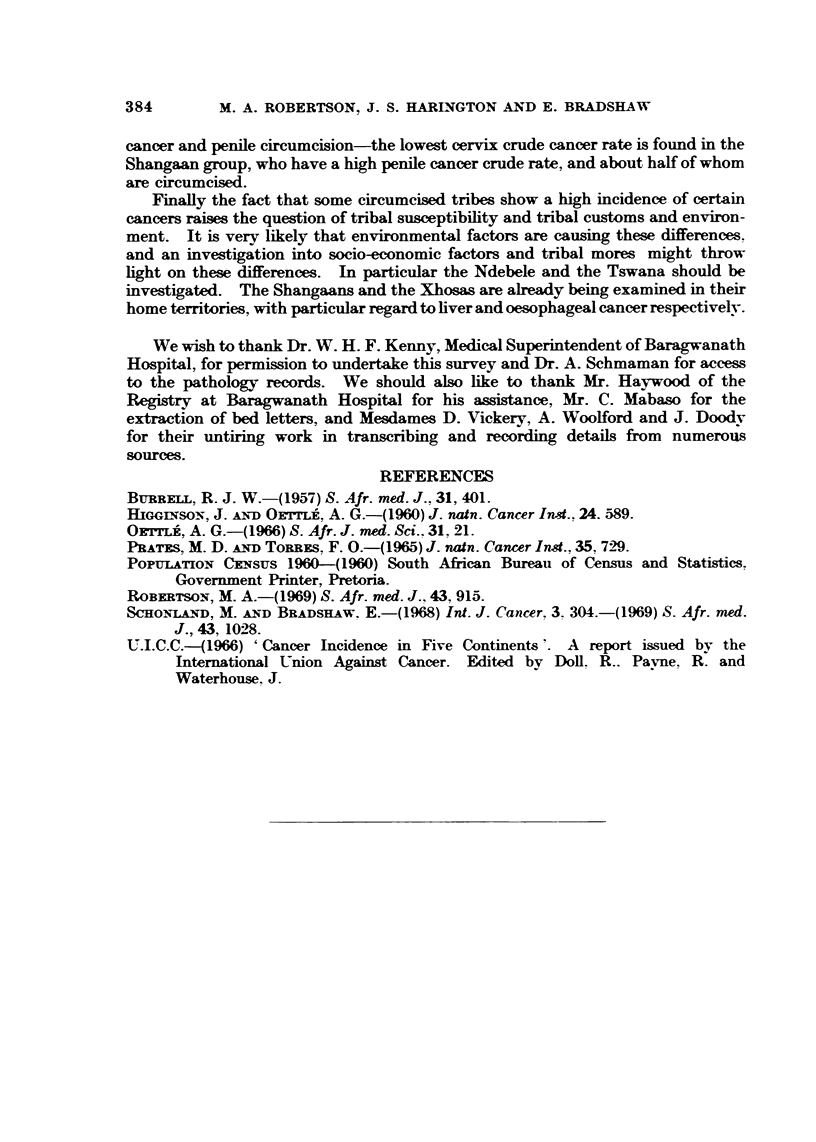# The Cancer Pattern in Africans at Bargwanath Hospital, Johannesburg†

**DOI:** 10.1038/bjc.1971.48

**Published:** 1971-09

**Authors:** M. A. Robertson, J. S. Harington, Evelyn Bradshaw

## Abstract

Material on African cancer cases admitted to Baragwanath Hospital, Johannesburg, over the years 1948-64 has been analysed, and it has been possible to obtain a useful incidence rate, a ratio study and a tribal analysis for purposes of comparison. The incidence rate, when compared to an earlier Johannesburg survey, showed a rise in oesophageal cancers for males and females. Both lung and prostate cancers showed rising rates in the men, while the female breast and cervix cancer rates remained relatively constant. Liver cancers had decreased in both sexes.


### THE CANCER PATTERN IN AFRICANS AT BARAGWANATH HOSPITAL, JOHANNESBURG†

M. A. ROBERTSON*, J. S. HARINGTON AND EVELYN BRADSHAW

*From the Cancer Research Unit of the National Cancer Association of South Africa, South African Institute for Medical Research, P.O. Box 1038, Johannesburg, South Africa*

Received for publication March 15, 1971

**SUMMARY.—Material on African cancer cases admitted to Baragwanath Hospital, Johannesburg, over the years 1948–64 has been analysed, and it has been possible to obtain a useful incidence rate, a ratio study and a tribal analysis for purposes of comparison. The incidence rate, when compared to an earlier Johannesburg survey, showed a rise in oesophageal cancers for males and females. Both lung and prostate cancers showed rising rates in the men, while the female breast and cervix cancer rates remained relatively constant. Liver cancers had decreased in both sexes.**

* Deceased, December 4, 1970.

† Reprints from Dr. J. S. Harington.

**The ratio study compared cancer admissions to the hospital for two periods, 1950–54 and 1960–64, and confirmed the rise in oesophageal and respiratory cancers in males, the constant proportion of breast cancers in females, and the tendency for liver cancers to lessen in recent years.**

**The tribal analysis showed differences in the distribution of cancer at specific sites among several tribes. In particular, further studies of the Xhosa, Tswana and Ndebele with high incidence and the Shangaan, with low incidence, would be fruitful.**

RECENT years have seen cancer registration developed in many places, and it is in the nature of geographical pathology that contributions offering new information and new aspects must be considered in relation to earlier studies in the same area, or similar studies carried out elsewhere. Since information on cancer among non-westernised peoples is scarce, and since incidence rates for Africans have only been available for relatively short periods (Oettlé, 1966; Schonland and Bradshaw, 1968; Prates and Torres, 1965), it is the intention of the present study to consider data on malignant neoplasms collected from a modern African hospital in Johannesburg, South Africa, and to compare this data with other studies on Africans in South Africa. This work was initiated by the late Dr. A. G. Oettlé.

*Baragwanath Hospital*

The Baragwanath Hospital is a large general hospital near Johannesburg in the Transvaal Province of South Africa, which is run on modern lines, and includes most of the specialised services. As it is situated in a rapidly developing area, the population served is consequently continually expanding, and the hospital has to cope with a general and constant increase in all departments. It was transferred from a military to a civilian non-white hospital in May, 1948. Approximately 800 beds were available when the hospital opened, and the number of beds has increased steadily to the stage where, in 1967, more than 2100 beds were in constant use. A previous study of the Baragwanath Hospital (Robertson, 1969) has drawn attention to clinical aspects of the cancer patterns at this hospital.

The population served is that of the non-white townships surrounding Johannesburg, excluding the miners working on the "Reef" mines as these attend mine hospitals. Most patients are from the immediate environment of Johannesburg, but, owing to the specialised departments available, some cases are referred from the outlying towns or rural districts of the Transvaal province.

*Hospital Admissions*

A survey of all hospital admissions for the months of March and October, 1964, excluding maternity cases, showed that 85·9% of patients were from Johannesburg and adjoining areas, 10·0% from local towns, 3·1% from rural areas, and 1·0% were unspecified. The race of the patients was in the ratio of 95·6% African, 3·3% Coloured, 0·8% Asiatics, and 0·3% not stated. Male admissions were greater than female admissions (59% and 41%), a reflection of the male predominance of the population served. The age distribution of the admissions showed that the bulk of patients were adults of working age (20–49 years), and children. The total admissions included paediatric, gynaecological, medical and surgical cases. Table I shows these features.

Registration of all cancer cases admitted to the hospital was carried out during the period 1948–64. Higginson and Oettlé (1960) used this material for the years 1953–55 in the first South African studies. All cases were checked to exclude duplications and were classified under the rubrics of the International Classification of Diseases (1957).

TABLE I.—*Admissions to Baragwanath Hospital (Excluding Maternity Cases)*

| | March 1964 % | October 1964 % | Average % |
|---|---|---|---|
| Total number of cases | 3138 | 3293 | 6431 |
| 1. *Domicile* | | | |
| Johannesburg metropolitan area | 85·5 | 86·2 | 85·9 |
| Johannesburg periphery | 10·2 | 9·9 | 10·0 |
| Transvaal towns | 3·4 | 2·8 | 3·1 |
| Transvaal rural | 0·9 | 1·1 | 1·0 |
| 2. *Race* | | | |
| African | 95·1 | 96·1 | 95·6 |
| Asiatic | 1·0 | 0·6 | 0·8 |
| Coloured | 3·6 | 3·0 | 3·3 |
| Not stated | 0·3 | 0·3 | 0·3 |
| 3. *Sex* | | | |
| Male | 57·4 | 60·1 | 58·8 |
| Female | 42·6 | 39·9 | 41·2 |
| 4. *Age* | | | |
| 0–4 | 10·4 | 9·1 | 9·7 |
| 5–19 | 13·6 | 14·3 | 14·0 |
| 20–29 | 25·1 | 23·3 | 24·2 |
| 30–39 | 21·1 | 20·7 | 20·9 |
| 40–49 | 14·4 | 15·2 | 14·8 |
| 50–59 | 6·9 | 8·2 | 7·6 |
| 60–69 | 4·4 | 4·9 | 4·6 |
| 70+ | 2·7 | 3·3 | 3·0 |
| Unknown | 1·4 | 1·0 | 1·2 |
| 5. *Ward* | | | |
| Medical | 29·0 | 31·7 | 30·4 |
| Surgical | 43·5 | 45·5 | 44·6 |
| Gynaecological | 13·6 | 10·3 | 11·8 |
| Paediatric | 13·9 | 12·5 | 13·2 |

*Cancer Cases as a Proportion of Hospital Admissions*

Table II shows a steady increase in the number of admissions and the number of cancer cases in the period under review, with an average of 1·3 cancer cases per 100 admissions. There were 7817 cancer cases diagnosed for the first time, consisting of 4093 males and 3724 females.

Histological confirmation was available in 84% of cases, and the diagnosis of the remaining 16% of cases was made on clinical or radiological evidence. Leukaemia cases were slightly under-estimated as haematological reports were not always available for scrutiny. Intraepithelial carcinoma of the skin, conjunctiva, oesophagus, cervix and other tissues were excluded. There were 13 cases which had dual cancer, and one case with triple primary cancer pathology occurring at the same time.

TABLE II.—*Cancer Cases as a Percentage of all Admissions (Including Maternity Cases) for the Years* 1948–64, *Baragwanath Hospital*

| | Total admissions M. and F. | Total cancers | Cancer cases as % of total admissions | Male | Female |
|---|---|---|---|---|---|
| 1948 | not available | 83 | — | 44 | 39 |
| 1949 | not available | 185 | — | 85 | 100 |
| 1950 | not available | 233 | — | 119 | 114 |
| 1951 | 26,100 | 240 | 0·9 | 129 | 111 |
| 1952 | 29,530 | 331 | 1·1 | 182 | 149 |
| 1953 | 29,620 | 392 | 1·3 | 195 | 197 |
| 1954 | 33,470 | 353 | 1·0 | 161 | 192 |
| 1955 | 37,616 | 348 | 0·9 | 168 | 180 |
| 1956 | 37,756 | 420 | 1·1 | 205 | 215 |
| 1957 | 39,671 | 481 | 1·2 | 254 | 227 |
| 1958 | 41,607 | 491 | 1·2 | 249 | 242 |
| 1959 | 47,678 | 561 | 1·2 | 283 | 278 |
| 1960 | 46,842 | 594 | 1·3 | 305 | 289 |
| 1961 | 48,599 | 678 | 1·4 | 364 | 314 |
| 1962 | 52,974 | 744 | 1·4 | 403 | 341 |
| 1963 | 51,936 | 793 | 1·5 | 439 | 354 |
| 1964 | 55,612 | 890 | 1·6 | 508 | 382 |
| | 579,021 | 7,817 | 1·3 | 4,093 | 3,724 |

The 7817 cancers registered in the period 1948–64 were analysed by site and sex and this showed that the most common male cancer sites are oesophagus, liver, buccal cavity and pharynx, and lung, in that order, while in females, cancers of the cervix and breast accounted for more than half the cancers diagnosed.

*Incidence Rates*, 1958–62

Because of the wide area served, the rapidly increasing population and the presence of three smaller hospitals in the neighbourhood, it is not possible to estimate an accurate Johannesburg African cancer incidence rate from material from Baragwanath hospital alone. However, it is possible, owing to the relatively large size of the hospital, to obtain a useful incidence rate, a ratio study, and a tribal analysis, from which comparisons can be made. In order to calculate age-adjusted incidence rates for Johannesburg, material pertaining to the years 1958–62 was selected, as a South African population census was taken in 1960. Only those cases with Johannesburg residential addresses were included. As the case finding programme did not include the other smaller hospitals in the area, this is an incomplete survey, and is probably an underestimate, though it is likely that the majority of cancer cases would pass through Baragwanath at some point in their illness. The age-adjusted cancer incidence rates per 100,000 population were calculated by using the annual mean number of cancer cases at specific sites, and the Johannesburg population for 1960 (Population Census, 1960), and then standardised against the African Standard Population (U.I.C.C., 1966). The standardised cancer incidence rates for the nine most common cancer sites in African males and females are shown in Table III, and these rates are compared with similar standardised cancer incidence rates for Johannesburg Africans (1953–55) (Oettlé, 1966).

From Table III we can make the following observations:

(1) The overall cancer incidence for Johannesburg Africans calculated from the Baragwanath cancer admissions for the period 1958–62 is very similar to the overall

TABLE III.—*Age-adjusted Cancer Incidence Rates for Baragwanath Patients, 1958–62, and Johannesburg Residents* 1953–55 (*per* 100,000 *Population*)

| | Males | | Females | |
|---|---|---|---|---|
| | Baragwanath | Johannesburg | Baragwanath | Johannesburg |
| All malignancies | 63·1 | 65·9 | 74·8 | 88·3 |
| Buccal cavity and pharynx | 4·2 | 4·2 | 1·1 | 1·4 |
| Oesophagus | 15·1 | 7·7 | 3·2 | 0·6 |
| Stomach, bowel and rectum | 4·2 | 8·0 | 3·4 | 7·4 |
| Liver | 7·8 | 13·5 | 2·2 | 6·1 |
| Lung | 5·2 | 4·6 | 1·2 | 1·7 |
| Breast | — | — | 8·5 | 9·5 |
| Cervix uteri | — | — | 29·7 | 35·6 |
| Prostate | 5·8 | 4·3 | — | — |
| Bladder | 1·6 | 2·1 | 0·6 | 0·6 |

cancer incidence found for Johannesburg Africans seven years previously, and confirms the validity of the earlier rates.

(2) In spite of the similarities in the overall incidence, the rate for oesophageal cancers has doubled in males and has risen five-fold in females, which confirms the rising trend noted in other surveys (Schonland and Bradshaw, 1969).

(3) The liver cancer rates in both sexes for the period 1958–62 is half that of the earlier survey. This is likely to represent a real decrease in the incidence of this cancer.

(4) In males, both lung and prostatic cancer rates have risen slightly, an increase that is in line with the high rates found in Durban (1964–66), (Schonland and Bradshaw, 1968).

(5) Rates for female breast cancer have barely altered.

(6) There is a decrease in the incidence of cancer of the cervix in the recent survey.

(7) The incidence of gastric and colonic cancer is lower in the recent survey.

TABLE IV.—*Baragwanath Ratio Study: Specific Site Cancers as a Percentage of all Cancers. Comparison Between* 1950–54 *and* 1960–64

| | | Males | | Females | |
|---|---|---|---|---|---|
| I.C.D. No. | Site | 1950–54 % | 1960–64 % | 1950–54 % | 1960–64 % |
| 140–8 | Buccal cavity and pharynx | 9·8 | 7·7 | 2·0 | 1·9 |
| 150 | Oesophagus | 10·3 | 27·5 | 0·8 | 4·7 |
| 151–4 | Stomach, bowel and rectum | 8·0 | 5·4 | 4·8 | 4·5 |
| 155 | Liver | 12·3 | 9·9 | 1·6 | 2·7 |
| 156–9 | Rest of G.I.T. | 3·4 | 2·8 | 2·0 | 2·1 |
| 160 | Nasal sinuses | 3·1 | 2·9 | 2·2 | 2·6 |
| 161–4 | Larynx, lung | 8·9 | 11·7 | | |
| 170 | Breast | — | — | 10·6 | 10·7 |
| 171 | Cervix uteri | — | — | 48·7 | 40·7 |
| 172–6 | Other female genital organs | — | — | 5·8 | 8·9 |
| 170–9 | Male breast and genital organs | 11·5 | 8·8 | — | — |
| 180–1 | Kidney and bladder | 4·6 | 3·6 | 2·1 | 2·0 |
| 190–1 | Melanoma and skin | 6·0 | 2·0 | 5·5 | 4·0 |
| 192–5 | Eye, brain, CNS and endocrine glands | 3·4 | 4·0 | 4·1 | 5·0 |
| 196–7 | Bone and connective tissue | 6·2 | 3·3 | 2·5 | 2·6 |
| 200–5 | Lymphatic and haematopoetic tissue | 9·7 | 6·5 | 5·1 | 4·3 |
| 198–9 | Unspecified | 2·8 | 3·9 | 2·2 | 3·3 |
| Total cases | | 786 | 2019 | 763 | 1680 |
| | | $\chi^2_{13}$ : 147·71 0·01 > $p$ | | $\chi^2_{14}$ : 47·12 0·01 > $p$ | |

(Female values 2·2 and 2·6 are bracketed to cover both Nasal sinuses and Larynx, lung.)

In general one concludes that where a drop in the rate is found from the previous survey, this may well be due to the underestimation caused by the incomplete case-finding programme. However, where a rise is recorded, this must indicate a real rise.

### *Ratio Study*

In order to determine whether changes in the African pattern of cancer at Baragwanath hospital occurred between the periods 1950–54 and 1960–64, cancer cases registered during these periods were compared on a ratio study basis. The method involved sorting the cancers for each 5-year period by site into 14 groups for the men, and 15 groups for the women, calculating specific site cancers as a percentage of all cancers, by sex, and comparing the two periods. Table IV gives this comparison. Differences were found in the distribution of cancers within the two periods for both sexes, and these differences were significant at the 1% level.

It will be noted that in African males oesophagus cancer (150) had risen from 10·3% to 27·5% of all cancers, and respiratory cancers (161–4) had risen from 8·9% to 11·7%. Liver cancer (155) formed a lower percentage of all cancers in 1960–64 than in 1950–54. In African females there was a rise in oesophagus cancers (150) also (0·8% to 4·7%), although the proportion of breast cancers remained exactly the same and liver cancers showed little change. The unchanging incidence of breast cancer in the two periods (and in the age-adjusted incidence rates) suggests that the breast cancer pattern in the women is not changing although cancers at other sites are varying (*e.g.* oesophagus cancers rising, cervix and skin cancers diminishing).

### *Tribal Distribution of Cancers*

The cancer patients came from many different tribes (Zulu, Sotho, Xhosa, Tswana, Swazi, Shangaan, Ndebele and others), but it appeared that there was an undue frequency of certain cancers in certain tribes, suggesting that there might be tribal differences in cancer incidence (chi-square tests were significant). It was thought necessary to relate this to the numbers of each tribe seen at the hospital, and the probable tribal distribution of admissions to Baragwanath was estimated for the period 1960–64.

Crude rates per 1000 admissions were calculated by sex for cancers of specific rates, for the tribes separately, for the period 1960–64.* These rates are in no

TABLE V.—*High and Low Cancer Incidence in Tribes*

| | All cancers | | Mouth | | Oesophagus | | Liver | | Nasal sinus | | Breast | Cervix | Penis | Lymphatic + haemic | |
|---|---|---|---|---|---|---|---|---|---|---|---|---|---|---|---|
| | M | F | M | F | M | F | M | F | M | F | F | F | M | M | F |
| Zulu | .. | .. | — | .. | .. | .. | .. | .. | .. | .. | .. | .. | .. | .. | .. |
| Sotho | .. | + | .. | .. | .. | .. | .. | .. | .. | .. | .. | + | .. | .. | .. |
| Xhosa | .. | .. | + | .. | + | .. | + | .. | .. | .. | .. | .. | .. | .. | .. |
| Tswana | + | + | + | + | + | .. | .. | .. | + | .. | .. | + | .. | .. | .. |
| Swazi | .. | .. | .. | + | .. | .. | .. | .. | .. | .. | .. | .. | .. | .. | .. |
| Shangaan | — | — | .. | — | — | — | + | + | .. | .. | .. | — | + | + | — |
| Ndebele | + | + | .. | — | + | + | .. | + | — | .. | .. | + | + | + | + |
| Other | .. | .. | .. | .. | .. | .. | .. | .. | .. | .. | .. | .. | .. | .. | .. |

* These rates are not included in this paper and are available on request.

sense comparable with standardised cancer incidence rates, and have been calculated to form a rough estimate of tribal susceptibilities and in Table V we show the high and low rates only for certain common cancers.

From this table it appears that the Tswana and Ndebele of both sexes have a high overall cancer incidence, as do Sotho women; whereas Shangaans of both sexes have a low incidence.

With the exception of cervix cancer in the Sotho women which is very high, and mouth cancer in the Swazi women, which was also high, the Zulus, Sothos and Swazis form the average group of cancer incidence.

The Xhosa males have a relatively high incidence of mouth, oesophageal and liver cancers, the last two being found again in Xhosas from the Transkei on the gold mines. The high oesophageal rate is well known and was first demonstrated by Burrell (1957) in the Transkei.

The high cancer incidence in Tswana males comes from high rates for mouth, oesophagus and nasal sinus cancers which may be aetiologically inter-related. The Tswana females have a high incidence of mouth and cervix cancers. It is of interest that a raised incidence of mouth cancer is found in both sexes.

Shangaans of both sexes have a very low overall cancer incidence, but again in both sexes, a high incidence of liver cancer is found. These Shangaans, although living in Johannesburg, originally came from Mozambique, which has the highest incidence rate of liver cancer in the world (U.I.C.C., 1966).

Of particular interest is the very high rate found in the Ndebele, a tribe of whom the Southern group live some 40 miles east of Johannesburg. Both sexes show a very high incidence of oesophageal cancer, approximating in this study to the rate found for Xhosas in Johannesburg. They also show a high rate of haemo-lymphoreticular malignancies in both sexes. The incidence of both cervix and penis cancers is raised.

It is generally considered that penile circumcision prevents penile cancer, but among those people practising penile circumcision there is no constant association with a low cervix cancer rate. In Table VI we show male circumcision practices

TABLE VI.—*Male Circumcision Practices by Tribe in Relation to Penile and Cervix Cancer Crude Rates (per* 1000 *Hospital Admissions)*

| Tribe | Male circumcision | Penile carcinoma | Carcinoma of cervix |
|---|---|---|---|
| Zulu | — | 0·7 | 7·5 |
| Sotho | ± | 0·4 | 12·8 |
| Xhosa | + | 0·3 | 6·4 |
| Tswana | — | 0·8 | 13·7 |
| Swazi | — | 0·3 | 10·8 |
| Shangaan | ± | 0·9 | 3·1 |
| Ndebele | — | 1·0 | 11·3 |

+ = 80% or more males are circumcised.
— = 20% or less males are circumcised.
± = practice varies within the group.

by tribe in relation to penile and cervix crude cancer rates. (Information on circumcision was derived from interviews on African male patients at Baragwanath during the period 1960–64.) Xhosas, who do circumcise, have a low penile crude cancer rate, whereas those tribes who do not, tend to have higher rates, with the exception of the Swazis. There is no constant association between cervix cancer and penile circumcision—the lowest cervix crude cancer rate is found in the Shangaan group, who have a high penile cancer crude rate, and about half of whom are circumcised.

Finally the fact that some circumcised tribes show a high incidence of certain cancers raises the question of tribal susceptibility and tribal customs and environment. It is very likely that environmental factors are causing these differences, and an investigation into socio-economic factors and tribal mores might throw light on these differences. In particular the Ndebele and the Tswana should be investigated. The Shangaans and the Xhosas are already being examined in their home territories, with particular regard to liver and oesophageal cancer respectively.

We wish to thank Dr. W. H. F. Kenny, Medical Superintendent of Baragwanath Hospital, for permission to undertake this survey and Dr. A. Schmaman for access to the pathology records. We should also like to thank Mr. Haywood of the Registry at Baragwanath Hospital for his assistance, Mr. C. Mabaso for the extraction of bed letters, and Mesdames D. Vickery, A. Woolford and J. Doody for their untiring work in transcribing and recording details from numerous sources.

### REFERENCES

Burrell, R. J. W.—(1957) *S. Afr. med. J.*, **31**, 401.

Higginson, J. and Oettlé, A. G.—(1960) *J. natn. Cancer Inst.*, **24**, 589.

Oettlé, A. G.—(1966) *S. Afr. J. med. Sci.*, **31**, 21.

Prates, M. D. and Torres, F. O.—(1965) *J. natn. Cancer Inst.*, **35**, 729.

Population Census 1960—(1960) South African Bureau of Census and Statistics, Government Printer, Pretoria.

Robertson, M. A.—(1969) *S. Afr. med. J.*, **43**, 915.

Schonland, M. and Bradshaw, E.—(1968) *Int. J. Cancer*, **3**, 304.—(1969) *S. Afr. med. J.*, **43**, 1028.

U.I.C.C.—(1966) 'Cancer Incidence in Five Continents'. A report issued by the International Union Against Cancer. Edited by Doll, R., Payne, R. and Waterhouse, J.